# The Complex Effects of PKM2 and PKM2:IP_3_R Disruption on Intracellular Ca^2+^ Handling and Cellular Functions

**DOI:** 10.3390/cells12212527

**Published:** 2023-10-26

**Authors:** Fernanda O. Lemos, Ian de Ridder, Martin D. Bootman, Geert Bultynck, Jan B. Parys

**Affiliations:** 1 Laboratory of Molecular and Cellular Signaling, Department of Cellular and Molecular Medicine & Leuven Kanker Instituut, KU Leuven, Herestraat 49, Campus Gasthuisberg O&NI—B802, 3000 Leuven, Belgium; ian.deridder@kuleuven.be (I.d.R.); geert.bultynck@kuleuven.be (G.B.); 2 School of Life, Health and Chemical Sciences, The Open University, Walton Hall, Milton Keynes MK7 6AA, UK; martin.bootman@open.ac.uk

**Keywords:** pyruvate kinase M2, IP_3_ receptor, intracellular Ca^2+^ signaling, apoptosis, ER-mitochondria contact sites

## Abstract

Pyruvate kinase M (PKM) 2 was described to interact with the inositol 1,4,5-trisphosphate (IP_3_) receptor (IP_3_R) and suppress its activity. To further investigate the physiological importance of the PKM2:IP_3_R interaction, we developed and characterized HeLa PKM2 knockout (KO) cells. In the HeLa PKM2 KO cells, the release of Ca^2+^ to the cytosol appears to be more sensitive to low agonist concentrations than in HeLa wild-type (WT) cells. However, upon an identical IP_3_-induced Ca^2+^ release, Ca^2+^ uptake in the mitochondria is decreased in HeLa PKM2 KO cells, which may be explained by the smaller number of contact sites between the ER and the mitochondria. Furthermore, in HeLa PKM2 KO cells, mitochondria are more numerous, though they are smaller and less branched and have a hyperpolarized membrane potential. TAT-D5SD, a cell-permeable peptide representing a sequence derived from IP_3_R1 that can disrupt the PKM2:IP_3_R interaction, induces Ca^2+^ release into the cytosol and Ca^2+^ uptake into mitochondria in both HeLa WT and PKM2 KO cells. Moreover, TAT-D5SD induced apoptosis in HeLa WT and PKM2 KO cells but not in HeLa cells completely devoid of IP_3_Rs. These results indicate that PKM2 separately regulates cytosolic and mitochondrial Ca^2+^ handling and that the cytotoxic effect of TAT-D5SD depends on IP_3_R activity but not on PKM2. However, the tyrosine kinase Lck, which also interacts with the D5SD sequence, is expressed neither in HeLa WT nor PKM2 KO cells, and we can also exclude a role for PKM1, which is upregulated in HeLa PKM2 KO cells, indicating that the TAT-D5SD peptide has a more complex mode of action than anticipated.

## 1. Introduction

Intracellular Ca^2+^ signals regulate a plethora of cellular processes, including fertilization, proliferation, differentiation, secretion, metabolism, contraction, motility, gene transcription, and cell fate. In almost all cell types, these Ca^2+^ signals depend primarily on the activity of the inositol 1,4,5-trisphosphate (IP_3_) receptor (IP_3_R), a Ca^2+^-release channel located in the endoplasmic reticulum (ER) [1]. IP_3_-induced Ca^2+^ release depends on the production of IP_3_ by phospholipase C, which most often occurs subsequent to agonist binding to G-protein-coupled receptors or receptors coupled to tyrosine kinase activity [2,3,4]. Importantly, the spatio-temporal characteristics of the eventual Ca^2+^ signals, as well as the subsequent cellular response, depend a great deal on the regulation of the subcellular localization and activity level of the IP_3_R. These are controlled by a combination of cytosolic factors including Ca^2+^ and ATP, via post-translational processes such as phosphorylation [5], and through direct interactions with regulatory or structural proteins [6,7,8].

Although the number of potential IP_3_R interactors already exceeds 150 described proteins [6], novel interacting proteins are still emerging. Of these novel proteins, an especially interesting protein is protein kinase M (PKM) 2. PKM2 is expressed in several normal adult tissues, though especially high levels of expression are found in embryonic tissues as well as in most tumor cells, where its expression level correlates with a poor clinical outcome [9,10,11,12].

PKM2 controls the conversion of phosphoenolpyruvate into pyruvate and ATP, the rate-limiting step of the glycolysis pathway, but it also performs several non-metabolic functions. These include the regulation of transcription via various mechanisms and the phosphorylation of various proteins in the cytosol, nucleus, and mitochondria [10,13]. Moreover, it was recently described that PKM2 modulates IP_3_R expression levels [14] and suppresses IP_3_-induced Ca^2+^ release [15] in several cell types.

In the present study, using a novel PKM2 knockout (KO) model (HeLa PKM2 KO cells) and the TAT-D5SD peptide previously described to disrupt the PKM2:IP_3_R complex [15], we investigated the physiological role of the PKM2:IP_3_R complex in mitochondrial function and cell death. Our results indicate complex effects of PKM2 on mitochondrial Ca^2+^ handling and mitochondrial metabolism. Moreover, we demonstrate that the TAT-D5SD peptide induces apoptosis in an IP_3_R-dependent way, yet independently from PKM2, PKM1, and the tyrosine kinase Lck. These results indicate a more complex impact of the peptide on cell survival beyond disrupting the PKM2:IP_3_R complex.

## 2. Materials and Methods

### 2.1. Peptides and Antibodies

The peptides D5SD, TAT-D5SD, Ctrl, and TAT-Ctrl were synthesized by Lifetein as previously described [15]. The following primary antibodies were used in this study: PKM1/2 (Cell Signaling Technology, Danvers, MA, USA, Cat. 3190); PKM1 (ProteinTech, Rosemont, IL, USA, Cat. 15821-1-AP); PKM2 (Cell Signaling Technology, Cat. 4053); IP_3_R1 (Rbt03 [16]); IP_3_R2 (Abiocode, Agoura Hills, CA, USA, Cat. R2872-3); IP_3_R3 (BD Pharmingen, Franklin Lakes, NJ, USA, Cat. 610213); SERCA2 (Cell Signaling Technology, Cat. 9580); ß-actin (Sigma, St. Louis, MO, USA, Cat. A-5441); PARP (Cell Signaling Technology, Cat. 9532); and Lck (Santa Cruz Biotechnology, Dallas, TX, USA, Cat. sc-433).

### 2.2. Cell Culture, including the Generation of PKM2 KO Cells and the Downregulation of PKM1

Wild-type HeLa (HeLa WT) and HeLa 3KO cells which expressed neither of the three IP_3_R isoforms [17] were cultured as previously described [15]. HeLa PKM2 KO cell lines were generated using the sequences ATTTGAGGAACTCCGCCGCC and GGCGGCGGAGTTCCTCAAATC, which were designed based on exon 10 of the PKM gene, and inserted into plasmid pSpCas9(BB)-2A-Puro (PX459) V2.0, which was a gift from Feng Zhang (Addgene, Watertown, MA, USA, plasmid # 62988) [18]. The HeLa WT cells were transfected with 1 µg of the plasmid and 3 µL of the transfection reagent X-treme Gene HP DNA (Roche, Basel, Switzerland, Cat. # 06366236001). Transfected cells were selected using 3 µg/mL of puromycin (Invivo Gen, San Diego, CA, USA, Cat. ant-pr) for 3 consecutive days to allow for complete PKM2 knockout. For the downregulation of PKM1, HeLa PKM2 KO cells previously plated on four-chamber 35 mm glass-bottom dishes (Cellvis, Mountain View, CA, USA, Cat. D35C4-20-0-N) were transfected with 20 nM of siRNA targeting exon 6 of human PKM mRNA (hs.Ri.PKM.13.1, Integrated DNA Technologies, Leuven, Belgium) or 20 nM of non-targeting control pool siRNA (Dharmacon/Horizon Discovery, Cambridge, UK). The transfection reagent Lipofectamine RNAiMAX (Invitrogen, Waltham, MA, USA, Cat. L3000) was used according to the manufacturer’s instructions. At 48 h post transfection, the cells were used for experiments. Effective PKM1 knockdown was confirmed via Western blotting. All cell lines were routinely tested for contamination with mycoplasma.

### 2.3. SDS-PAGE, Western Blot Analysis, and Immunoprecipitation Experiments

HeLa cells were washed twice with ice-cold phosphate-buffered saline (PBS), incubated for 30 min in ice-cold RIPA lysis buffer (20 mM Tris; 150 mM NaCl; 1.5 mM MgCl_2_; 0.5 mM dithiothreitol; 0.01% Triton X-100; protease inhibitors (Thermo Fisher Scientific, Waltham, MA, USA, Cat. A32965)), and centrifuged for 5 min at 14,000 rpm at 4 °C. Lysates containing 10–20 μg of protein were separated on NuPAGE 4–12% Bis/Tris SDS-polyacrylamide gels (Thermo Fisher Scientific, Cat. NP0322), using a MOPS/SDS-running buffer (Thermo Fisher Scientific, Cat. NP000102), and transferred onto a PVDF Transfer Membrane, 0.45 µm (Thermo Fisher Scientific, Cat. 88518). The membranes were blocked with Tris-buffered saline (TBS) containing 0.1% Tween and 5% non-fat dry milk powder and incubated overnight with the primary antibody. Next, the membranes were incubated for 1 h with anti-mouse HRP-conjugated secondary antibody (Bioke, Leiden, The Netherlands, Cat. 7076) or anti-rabbit, light-chain-specific HRP-conjugated secondary antibody (Jackson Immuno Research Laboratories, Cat. 211-032-171) diluted 1:2000 in 0.1% Tween/1% non-fat dry milk/TBS. Protein detection was performed using a Pierce ECL Western Blotting Substrate (Thermo Fisher Scientific, Cat. 32106) or a Clarity™ Western ECL substrate (Bio-Rad, Hercules, CA, USA, Cat. # 170-5061). The membrane was revealed in the ChemiDoc™ MP imaging system (Bio-Rad), and band quantification was performed using Image Lab 6.1 software (Bio-Rad).

For the immunoprecipitation experiments, HeLa WT cells were lysed using the procedure described above, except that an ice-cold CHAPS lysis buffer (50 mM Tris-HCl; 100 mM NaCl; 2 mM EDTA; 1% CHAPS; 50 mM NaF; 1 mM Na_3_VO_4_) containing protease inhibitors (Thermo Fisher Scientific, Cat. A32965) was used. Lysates (1.0 mg in 1 mL volume per sample) were incubated with gentle rotation with the D5SD or Ctrl peptide (200 µM) for at least 1 h at 4 °C. The mixture of cell lysate and peptide was further incubated overnight at 4 °C with gentle rotation, using Dynabeads (Life Technologies, Carlsbad, CA, USA, Cat. 10003D) pre-coated with 0.6 µg of an antibody against PKM2 (Cell Signaling Technology, Cat. 4053) or 0.6 µg of normal rabbit control IgG (SinoBiological, Beijing, China, Cat. CR1). The beads were washed 3× with ice-cold PBS, and the proteins were eluted via incubation in a 50 mM Tris buffer containing 0.2% SDS and 0.1% Tween for 30 min at room temperature and under gentle agitation.

### 2.4. Single-Cell Cytosolic Ca^2+^ Measurements

Cells plated on four-chamber 35 mm glass-bottom dishes (Cellvis, Cat. D35C4-20-0-N) were loaded for 30 min at room temperature with 1.25 µM of Fura-2-AM (AnaSpec, Fremont, CA, USA, Cat. AS-84017) diluted in a modified Krebs solution (150 mM NaCl, 5.9 mM KCl, 1.2 mM MgCl_2_, 11.6 mM HEPES (pH 7.3), 11.5 mM glucose, and 1.5 mM CaCl_2_). The cells were washed with the modified Krebs solution, and the incubation was continued for 30 min at room temperature to allow for the de-esterification of the Fura-2 AM. Immediately before the Ca^2+^ measurements, EGTA in a modified Krebs solution without Ca^2+^ (150 mM NaCl, 5.9 mM KCl, 1.2 mM MgCl_2_, 11.6 mM HEPES (pH 7.3), and 11.5 mM glucose) was added to a final concentration of 3 mM. ATP and peptides were diluted into the modified Krebs solution without Ca^2+^, supplemented with 3 mM of EGTA, and gently added to the chamber after basal acquisition. To measure the ER Ca^2+^-store content, thapsigargin (1 µM) was added to the medium. As thapsigargin is an irreversible inhibitor of the sarco/endoplasmic reticulum (ER) Ca^2+^-ATPase (SERCA) [19], it uncovers the basal Ca^2+^-leak pathway of the ER. The amount of Ca^2+^ subsequently released into the cytosol therefore represents the content of the ER Ca^2+^ store. To analyze store-operated Ca^2+^ entry (SOCE), 5 mM of CaCl_2_ was added to the medium after the Ca^2+^ signal triggered by thapsigargin reached basal levels again. An Axio Observer Z1 fluorescent microscope (Zeiss, Jena, Germany) was used to acquire the images. The Ca^2+^ signal was quantified by alternately exciting the Ca^2+^ indicator at 340 and 380 nm and collecting the fluorescence emitted at >510 nm. The images were analyzed using Fiji (National Institutes of Health). The data were plotted as F_340_/F_380_, and the baseline was measured for 60 s before adding the compound. Ca^2+^ traces were analyzed using Excel (Microsoft, Redmond, WA, USA) and Prism 9.1.0 (GraphPad, San Diego, CA, USA) software. The area under the curve (AUC) was calculated by integrating the responses to the agonist, using Graphpad Prism 9.1.0 software. For the calculation of the AUC, only the part of the response above the baseline was taken into consideration. The amplitude was calculated by subtracting the baseline from the maximal response to the agonist.

### 2.5. Single-Cell Mitochondrial Ca^2+^ Uptake Measurements

Cells plated on four-chamber 35 mm glass-bottom dishes (Cellvis, Cat. D35C4-20-0-N) were transfected with 0.5 µg/dish of pCMV R-CEPIA3mt, a gift from Dr. Masamitsu Iino (Addgene, plasmid # 140464) [20]. X-tremeGene HP DNA (Roche, Cat. # 06366236001) was used as a transfection reagent in accordance with the manufacturer’s instructions. Forty-eight hours after transfection, the cell medium was replaced by the modified Krebs solution. Immediately before the Ca^2+^ measurements, EGTA was added to a modified Krebs solution without Ca^2+^ to reach a final concentration of 3 mM. ATP, thapsigargin, and peptides were diluted into a modified Krebs solution without Ca^2+^, supplemented with 3 mM of EGTA, and gently added to the chamber after basal acquisition. To analyze mitochondrial uptake due to SOCE, 5 mM of CaCl_2_ was added to the medium after 10 min of the acquisition of the Ca^2+^ signal triggered by thapsigargin. An Axio Observer Z1 fluorescent microscope (Zeiss) was used to acquire the images using a 543/22 nm filter. The images were analyzed using Fiji. The data were plotted as F/F_0_ where F = F at different time points and F_0_ = the average of the baseline, measured for 60 s, before the compound was added. Ca^2+^ traces were analyzed as described above in Section 2.4.

### 2.6. Mitochondrial Morphology Analysis Using MitoTracker^TM^ Green

Cells previously plated on four-chamber 35 mm glass-bottom dishes (Cellvis, Cat. D35C4-20-0-N) were incubated for 15 min at room temperature with 100 nM of MitoTracker^TM^ Green (Invitrogen, Cat. M7514) diluted in a modified Krebs solution. After incubation, the cells were washed twice with the modified Krebs solution for live-cell imaging. The images were acquired in Z-stack mode with 0.3 µm/slide, using a Zeiss Axiovert 100M LSM 510 confocal microscope (Zeiss, Jena, Germany) equipped with a 63×/0.75 air objective (Nikon, Tokyo, Japan) and an 488 nm Argon laser equipped with an LP 505 filter. The quantification of organelle contact sites was performed using Fiji and the Mitochondria Analyzer plugin [21]. The following mitochondrial morphology parameters were quantified: the total mitochondrial volume/cell, the individual mitochondrial volume, the number of mitochondria per cell, and the number of branches per mitochondrion.

### 2.7. Quantification of ER-Mitochondria Contact Sites, Using the Split-GFP-Based Contact Site Sensor (SPLICS)

Cells previously plated on four-chamber 35 mm glass-bottom dishes (Cellvis, Cat. D35C4-20-0-N) were transfected with 2.0 µg/dish of SPLICS Mt-ER Short P2A plasmid, a gift from Dr. Tito Cali [22,23]. X-tremeGene HP DNA (Roche, Cat. # 06366236001) was used as a transfection reagent in accordance with the manufacturer’s instructions. Forty-eight hours after transfection, the cells were rinsed 3× with PBS and fixed with 4% paraformaldehyde for 15 min at room temperature. The cells were washed 3× with PBS and kept in PBS for acquiring the images. The images were acquired in Z-stack mode on a 0.3 µm/slide, using a Zeiss Axiovert 100M LSM 510 confocal microscope equipped with a 63×/0.75 air objective (Nikon), and an 488 nm Argon laser equipped with an LP 505 filter. The quantification of organelle contact sites was performed using Fiji, following a published protocol [23].

### 2.8. Measurement of Mitochondrial Membrane Potential (ΔΨ)

Cells previously plated on four-chamber 35 mm glass-bottom dishes (Cellvis, Cat. D35C4-20-0-N) were incubated for 15 min at room temperature with 2 µM of JC-1 (Invitrogen, Cat. T3168) or 10 nM of TMRE (Invitrogen, Cat. T669) diluted in a modified Krebs solution. After incubation, the cells were washed twice with the modified Krebs solution for live-cell imaging. For JC-1 staining, the images were acquired using a Zeiss Axiovert 100M LSM 510 confocal microscope equipped with a 63×/0.75 air objective (Nikon, Tokyo, Japan), an 488 nm Argon laser equipped with a BP 505-530 filter, and a 543 nm laser equipped with an LP 560 filter. The images were analyzed using Fiji. The quantification of ΔΨ was expressed as the ratio of the intensity of the fluorescence in the green and red channels. For TMRE, images were acquired using a Zeiss Axiovert 100M LSM 510 confocal microscope equipped with a 63×/0.75 air objective (Nikon) and a 543 nm laser equipped with an LP 560 filter. After the image acquisition, FCCP (20 µM) was added, and the cells were further incubated for 10 min before another image was acquired from the same frame. The images were analyzed using Fiji. The quantification of ΔΨ was expressed as difference of the intensity of the fluorescence before and after the addition of FCCP.

### 2.9. Measurement of Oxygen Consumption Rate (OCR) and Extracellular Acidification Rate (ECAR)

The OCR and ECAR were measured using an XFp extracellular analyzer (Seahorse Bioscience, North Billerica, MA, USA). The cells were plated on XFp Cell culture miniplates (Seahorse Bioscience, Cat. 103022-100) at 15,000/well. The next day, the medium was changed to XF Base Medium (Seahorse Bioscience, Cat. 103334-100) containing 10 mM of glucose, and the cells were incubated at 37 °C in a non-CO_2_ incubator for 1 h. The cells were sequentially exposed to oligomycin (1 µM), FCCP (0.5 µM), and a mixture of rotenone (0.5 µM) and antimycin A (0.5 µM). The results were analyzed using the online software Seahorse Analytics (https://seahorseanalytics.agilent.com/Account/Login, accessed on 7 December 2022).

### 2.10. Cell Death Assays

For caspase-3 activity assays, HeLa WT, HeLa PKM2 KO, and HeLa 3KO cells were plated on black 96-well plates (Greiner, Kremsmünster, Austria, Cat. 655077) at 10,000 cells/well. The next day, the medium was removed, and the cells were washed twice with a medium. A medium containing 1:200 of NucView 488 caspase-3 assay reagent (Biotium, Fremont, CA, USA, Cat. 30029) was added, followed by the addition of the peptides (TAT-Ctrl or TAT-D5SD), which were diluted in the medium to a final concentration of 5 µM. The cells were automatically photographed every 2 h for 24 h using the IncuCyte ZOOM System at 10× magnification and the S3/SX1 G/R Optical Module, using phase contrast and a green channel (using 440–480 nm excitation, 504–544 nm emission, and a 300 ms acquisition time) and the images were analyzed using the software IncuCyte S3 (Sartorius, Göttingen, Germany), whereby the number of stained nuclei (green channel) were normalized to the confluence area (phase contrast).

For a PARP cleavage analysis, HeLa WT and HeLa PKM2 KO cells were plated on 6-well plates (VWR, Leuven, Belgium, Cat. 734-2323) at 300,000 cells/well. The next day, the cells were treated for 12 h with 10 µM of the peptides (TAT-Ctrl or TAT-D5SD) or with 0.5 µM of staurosporine. The cells were then harvested and lysed with RIPA buffer, as described above (see Section 2.1). Apoptosis was monitored via Western blotting in order to quantify cleaved and uncleaved PARP. PARP cleavage was calculated as the ratio of cleaved PARP over total PARP.

## 3. Results

### 3.1. PKM2 Negatively Modulates IP_3_R-Mediated Ca^2+^ Signal in HeLa Cells

**Figure 1:**
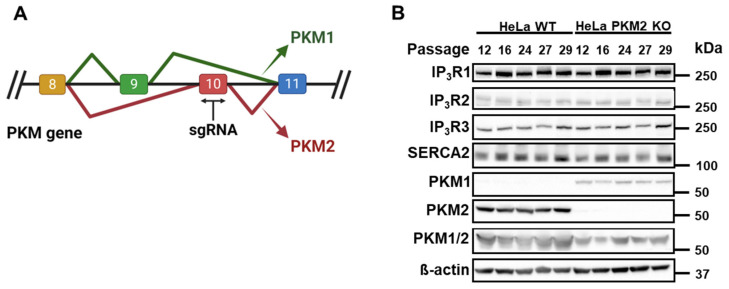
Characterization of HeLa PKM2 knockout (KO) cells. (**A**) Schematic representation of the PKM gene in which the single guide RNA (sgRNA) was selectively designed to target exon 10 in order to selectively knock out PKM2. (**B**) Western blot showing that compared to HeLa WT cells, HeLa PKM2 KO cells did not express any PKM2 while PKM1 was upregulated. Importantly, no alterations in the expression of the different isoforms of IP_3_R (IP_3_R1-3) or SERCA2 were observed. β-actin was used as a loading control. All primary antibodies were diluted to 1:1000. Cell lysates from five different passages are shown.

Our group previously showed that PKM2 interacts with IP_3_Rs and suppresses the IP_3_-dependent cytosolic release of Ca^2+^ in B cells and T cells, as well as in HeLa cells [15]. To extend our knowledge about the action of PKM2 on the IP_3_R’s activity, we generated HeLa PKM2 KO cells using a specific single guide RNA (sgRNA) based on the sequence of exon 10 of the PKM gene (Figure 1A). HeLa cells express PKM2 and the three isoforms of IP_3_Rs, and the interaction between them has already been described in this cell line [15]. As shown in Figure 1B, the exclusion splicing of PKM2 via genome editing was stable over several passages. Contrary to PKM1, which was upregulated in the HeLa PKM2 KO cells compared to the HeLa WT cells, immunoblotting for IP_3_R1, IP_3_R2, IP_3_R3, and SERCA2 showed that the absence of PKM2 did not affect the expression of these Ca^2+^ signaling proteins over time (Figure 1B). It is noteworthy that the upregulation of PKM1 occurring in the HeLa PKM2 KO cells did not compensate fully for the loss of PKM2. The measurement of the total PKM, obtained using an antibody that recognizes a conserved region between PKM1 and PKM2, was reduced in HeLa PKM2 knockout cells as the PKM/β-actin ratio decreased from 2.6 ± 0.7 in the HeLa WT cells to 1.2 ± 0.6 in the HeLa PKM2 KO cells (mean ± S.D., N = 5).

**Figure 2:**
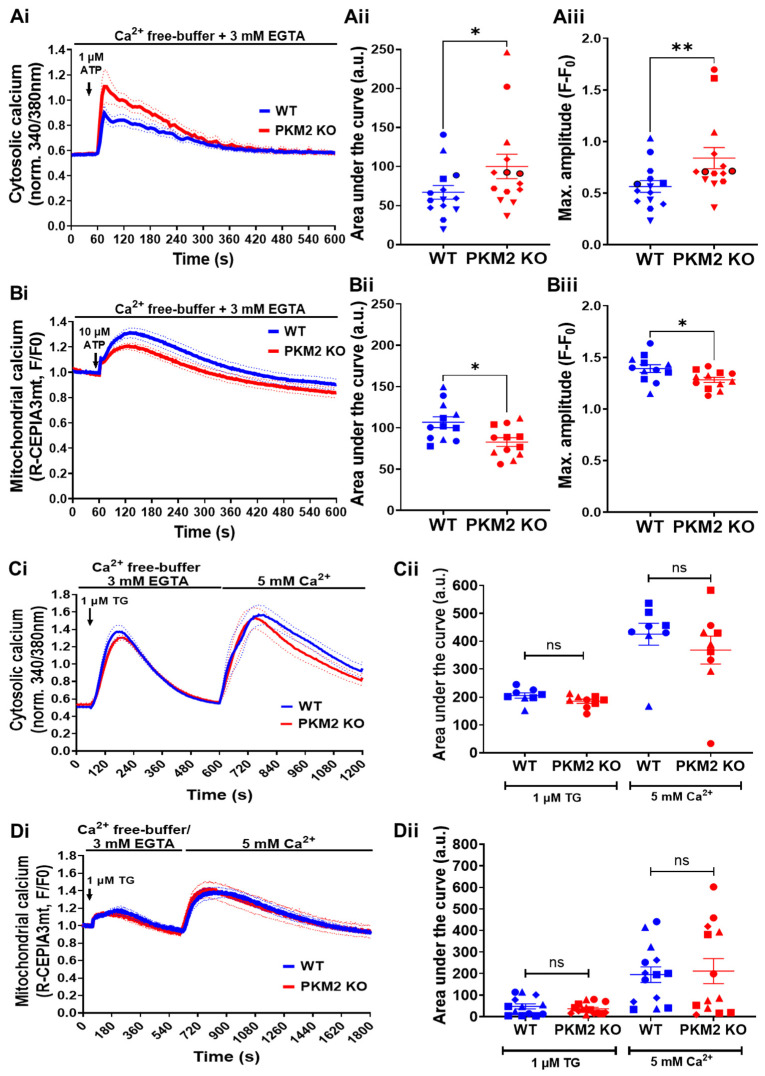
PKM2 affects IP_3_R-mediated intracellular Ca^2+^ signals in HeLa cells. (**Ai**) The time course of the IP_3_R-dependent cytosolic Ca^2+^ responses evoked in HeLa WT (blue) and HeLa PKM2 KO (red) cells. The single-cell Ca^2+^ signals were measured in the absence of extracellular Ca^2+^ in cells loaded with Fura-2 AM. As indicated by the arrow, 1 μM of ATP was added after 60 s of baseline measurement. The curves are presented as the mean (bold line) ± SEM (dashed line) values of seven independent experiments, each performed on 1-4 coverslips/experiments, with >25 cells/coverslip. (**Aii**) The quantification of the area under the curve and (**Aiii**) the maximal amplitude of the Ca^2+^ signals induced by 1 μM of ATP shown in Ai. Significance was analyzed using Student’s *t*-test (* *p* < 0.05; ** *p* < 0.01). (**Bi**) The time course of IP_3_R-dependent mitochondrial Ca^2+^ uptake in HeLa WT (blue) and HeLa PKM2 KO (red) cells transfected with R-CEPIA 3mt. After 48 h, mitochondrial Ca^2+^ signals were measured in the absence of extracellular Ca^2+^. As indicated by the arrow, 10 μM of ATP was added after 60 s of baseline measurement. The mitochondrial Ca^2+^ uptake was normalized to baseline fluorescence. The curves are presented as the mean (bold line) ± SEM (dashed line) values of rhree independent experiments, each performed on four coverslips/experiments, with ≥12 cells/coverslip. (**Bii**) The quantification of the area under the curve and (**Biii**) the maximal amplitude of the mitochondrial Ca^2+^ uptake induced by 10 μM of ATP shown in Bi. Significance was analyzed using Student’s *t*-test (* *p* < 0.05). (**Ci**) ER Ca^2+^ store content and store-operated Ca^2+^ entry into HeLa WT (blue) and HeLa PKM2 KO (red) cells. Cytosolic Ca^2+^ signals were measured in single cells loaded with Fura-2 AM. As indicated by the arrow, 1 μM of thapsigargin (TG) was added after 60 s of baseline measurement in the absence of extracellular Ca^2+^. After emptying the Ca^2+^ stores, CaCl_2_ (5 mM) was added to allow Ca^2+^ entry. The curves are presented as the mean (bold line) ± SEM (dashed line) values of three independent experiments, each performed on >35 cells/coverslip, with 2–3 coverslips/experiment. (**Cii**) The quantification of the area under the curve of the Ca^2+^ signals induced by thapsigargin or CaCl_2_, shown in Ci as mean ± SEM values. Significance was analyzed using Student’s *t*-test (ns = no statistical difference). (**Di**) Mitochondrial Ca^2+^ uptake caused by store-operated Ca^2+^ entry into HeLa WT (blue) and HeLa PKM2 KO (red) cells. As indicated by the arrow, 1 μM of thapsigargin (TG) was added after 60 s of baseline measurement in the absence of extracellular Ca^2+^ to cells previously transfected with R-CEPIA 3mt. After emptying the Ca^2+^ stores, CaCl_2_ (5 mM) was added to allow for Ca^2+^ entry. The curves are presented as the mean (bold line) ± SEM (dashed line) values of three independent experiments, each performed on >35 cells/coverslip, with 3–4 coverslips/experiment. (**Dii**) The quantification of the area under the curve of the mitochondrial Ca^2+^ uptake induced by thapsigargin or CaCl_2_, shown in Di as mean ± SEM values. Significance was analyzed using Student’s *t*-test (ns = no statistical difference).

We subsequently compared the IP_3_R-mediated Ca^2+^-signaling properties of Fura-2-loaded HeLa WT and HeLa PKM2 KO cells. To avoid the contribution of Ca^2+^ influx, the cells were treated with EGTA (3 mM) prior to and during ATP stimulation. Cytosolic Ca^2+^ was measured using Fura-2 after the treatment of the cells with 1 µM of ATP. In agreement with the previously observed inhibitory effect of PKM2 on the IP_3_R’s activity [15], increases in cytosolic [Ca^2+^] were larger in the HeLa PKM2 KO cells than in the HeLa WT cells (Figure 2A). Mitochondrial Ca^2+^ was measured using R-CEPIA3mt [24]. To accurately evaluate the impact of PKM2 on mitochondrial Ca^2+^ handling, we used a higher agonist concentration (ATP, 10 µM) to ensure similar IP_3_-induced increases in cytosolic [Ca^2+^] in the HeLa WT and HeLa PKM2 KO cells (Supplementary Figure S1). Under these conditions, the mitochondrial Ca^2+^ signal was, however, smaller in the HeLa PKM2 KO cells than in the HeLa WT cells (Figure 2B), indicating the existence of additional effects at the level of the mitochondria. As differences in Ca^2+^ release can be due to differential filling of the ER Ca^2+^ stores, in a separate experiment, we applied thapsigargin (1 µM) to examine the ER’s Ca^2+^ content (Figure 2C). No difference in the ER Ca^2+^ store content was observed between the HeLa WT and HeLa PKM2 KO cells. In the same experiment, the re-addition of extracellular Ca^2+^ (5 mM) also indicated no differences in SOCE between the HeLa WT and HeLa PKM2 KO cells. The mitochondrial Ca^2+^ uptake subsequent to the release of the ER’s Ca^2+^ content after the addition of thapsigargin, as well as the uptake triggered by SOCE, were also identical in the HeLa WT and HeLa PKM2 KO cells (Figure 2D).

### 3.2. Analysis of Mitochondrial Characteristics and of ER-Mitochondria Contact Sites

The close apposition between the ER and mitochondria at the so-called ER-mitochondria contact sites allows for the efficient transfer of Ca^2+^ ions from the ER to the mitochondrial matrix. This Ca^2+^ flux is mediated on the ER side by the IP_3_R and at the outer mitochondrial membrane by the voltage-dependent anion channel (VDAC) [25,26,27,28,29,30].

**Figure 3:**
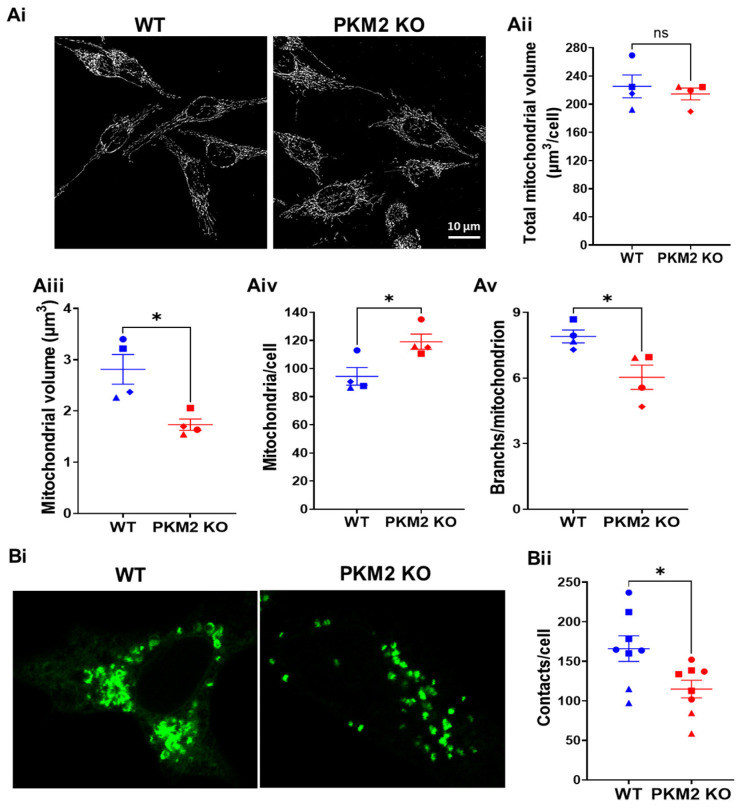
PKM2 alters mitochondrial morphology and increases ER-mitochondria contact. (**Ai**) Representative images of mitochondria in HeLa WT and HeLa PKM2 KO cells. Cells were stained with MitroTracker^TM^ Green (0.1 µM) for 15 min. Live-cell imaging was performed in z-stack mode, 0.3 µm/slide, in a confocal microscope. The following parameters of mitochondrial morphology were quantified: (**Aii**) total mitochondrial volume/cell; (**Aiii**) individual mitochondrial volume; (**Aiv**) number of mitochondria per cell; and (**Av**) number of branches per mitochondrion. Results are shown as the mean ± SEM values of 2–3 coverslips/experiment, with >10 cells/coverslips, in three independent experiments. Significance was analyzed using Student’s *t*-test (* *p* < 0.05; ns = no statistical difference). (**Bi**) Representative images of ER-mitochondria contacts in HeLa WT and HeLa PKM2 KO cells. Cells were transfected with the short variant of the split green fluorescent protein (GFP)-based contact site sensor (SPLICS (S)-P2A ER-MT) in order to detect ER-mitochondria proximity in the 8–10 nm range. After 48 h, the cells were fixed, and images were acquired in z-stack mode, 0.3 µm/slide, using a confocal microscope. (**Bii**) The quantification of ER-mitochondria contacts per cell, shown as the mean ± SEM values of 2–3 coverslips/experiment and ≥ 8 cells/coverslips in three independent experiments. Significance was analyzed using Student’s *t*-test (* *p* < 0.05).

To understand why at an equivalent level of Ca^2+^ signaling (Supplementary Figure S1), PKM2 KO decreased mitochondrial Ca^2+^ uptake (Figure 2B), we decided to compare the characteristics of the mitochondria in HeLa WT and HeLa PKM2 KO cells. In particular, it was important to assess whether the HeLa PKM2 KO cells contained the same global volume of mitochondria as the HeLa WT cells. An analysis of MitoTracker^TM^-Green-stained mitochondria indicated that although the total mitochondrial volume was identical between the two cell lines (WT: 225 ± 16 μm^3^/cell; PKM2 KO: 215 ± 8 μm^3^/cell), the mitochondria in the PKM2 KO cells were smaller (WT: 2.8 ± 0.3 μm^3^; PKM2 KO: 1.7 ± 0.1 μm^3^) but more numerous (WT: 94 ± 6 per cell; PKM2 KO: 119 ± 5 per cell) and less branched (WT: 7.9 ± 0.3; PKM2 KO: 6.0 ± 0.6 branches/mitochondrion) (Figure 3A).

Subsequently, we investigated the occurrence of ER-mitochondria contact sites in the HeLa WT and HeLa PKM2 KO cells. To this end, we used the short variant of the SPLICS sensor to detect ER-mitochondria proximity in the 8-10 nm range [22] in HeLa WT and HeLa PKM2 KO cells (Figure 3B). The quantification of the data revealed a significantly higher number of ER-mitochondria contact sites in the HeLa WT cells compared to the HeLa PKM2 KO cells (WT: 166 ± 16 contact sites/cell; PKM2 KO: 115 ± 11 contact sites/cell).

**Figure 4:**
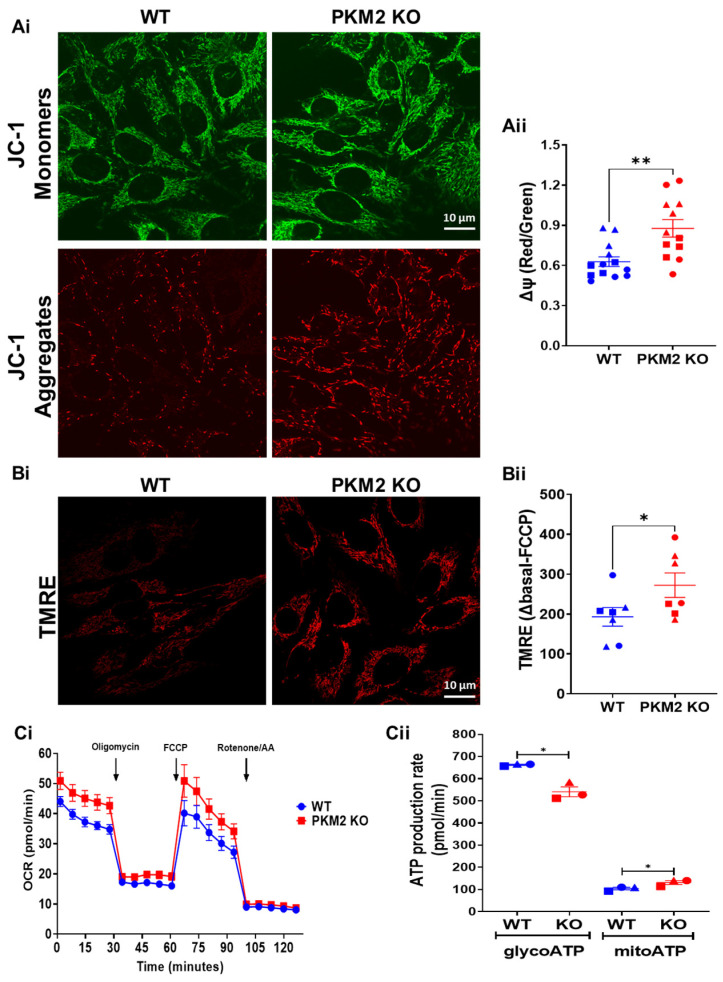
PKM2 reduces mitochondrial membrane potential and mitochondrial function in HeLa cells. Representative images of mitochondrial membrane potential analysis in HeLa WT and HeLa PKM2 KO cells. Cells were stained for 15 min with 2 µM of the ratiometric mitochondrial membrane potential (∆ψ) reporter JC-1 (**Ai**) or with 10 nM of TMRE (**Bi**). Live-cell imaging was performed using a confocal microscope. (**Aii**) The quantification of ∆ψ using the ratio of the JC-1 intensity of the images at 538 and 590 nm, shown as the mean ± SEM values of 4–5 coverslips/experiment with >9 cells/image in three independent experiments. (**Bii**) The quantification of ∆ψ using the difference in the intensity of TMRE under basal conditions and after 10 min of incubation with FCCP (20 µM), shown as the mean ± SEM values of three coverslips/experiment with >9 cells/images in three independent experiments. Significance was analyzed using Student’s *t*-test (* *p* < 0.05; ** *p* < 0.01). (**Ci**) The oxygen consumption rate (OCR) of the HeLa WT (blue) and HeLa PKM2 KO (red) cells in a medium supplemented with 10 mM of glucose. As indicated by the arrows, 1 µM of oligomycin, 0.5 µM of FCCP and a mixture of 0.5 µM of rotenone/0.5 µM of antimycin A (AA) were sequentially added to analyze mitochondrial function. The curves are presented as the mean ± SEM values of three independent experiments, each performed on three wells/group. (**Cii**) The quantification of glycolytic (glycoATP) and mitochondrial (mitoATP) ATP production in HeLa WT (blue) and HeLa PKM2 KO (red) cells, based on the results obtained in Ci. Results are shown as the mean ± SEM values of three independent experiments, each performed on three wells/group.

To further assess mitochondrial functionality, the mitochondrial membrane potential was determined, using either JC-1 (Figure 4A) or TMRE (Figure 4B). Both these approaches indicated a more negative potential in the HeLa PKM2 KO cells compared to the HeLa WT cells. Moreover, the HeLa PKM2 KO cells displayed a larger basal and maximal OCR, when compared to the HeLa WT cells (basal OCR—WT: 27.1 ± 1.3 pmol/min; PKM2 KO: 34.0 ± 2.1 pmol/min; maximal OCR—WT: 32.8 ± 4.7 pmol/min; PKM2 KO: 42.1 ± 4.7 pmol/min) (Figure 4Ci). Based on the OCR and ECAR, ATP production was calculated (Figure 4Cii). In the PKM2 KO cells, ATP production consequent to glycolysis was decreased (WT: 663 ± 3 pmol/min; PKM2 KO: 541 ± 22 pmol/min), while mitochondrial ATP production was slightly increased (WT: 104 ± 6 pmol/min; PKM2 KO: 131 ± 9 pmol/min).

### 3.3. Effects of the TAT-D5SD Peptide on Ca^2+^ Handling in HeLa WT and HeLa PKM2 KO Cells

To specifically investigate the regulatory role of PKM2 on IP_3_R-mediated Ca^2+^ signaling, in the absence of confounding effects due to indirect functions of PKM2, e.g., via the modulation of mitochondrial dynamics or function, we decided to directly act on the PKM2:IP_3_R interaction. For this, we used the D5SD peptide, a 21-amino-acid sequence corresponding to a.a. 2078–2098 of IP_3_R1 that is conserved among the three IP_3_R isoforms. This peptide, as well as its cell-permeable variant TAT-D5SD, a D5SD peptide fused at its N terminus to the HIV TAT sequence, can disrupt the PKM2:IP_3_R interaction in various cell lines [15]. In present study, our aim was to investigate the effect of PKM2:IP_3_R disruption on both cytosolic and mitochondrial Ca^2+^ handling, as well as on cell death induction.

**Figure 5:**
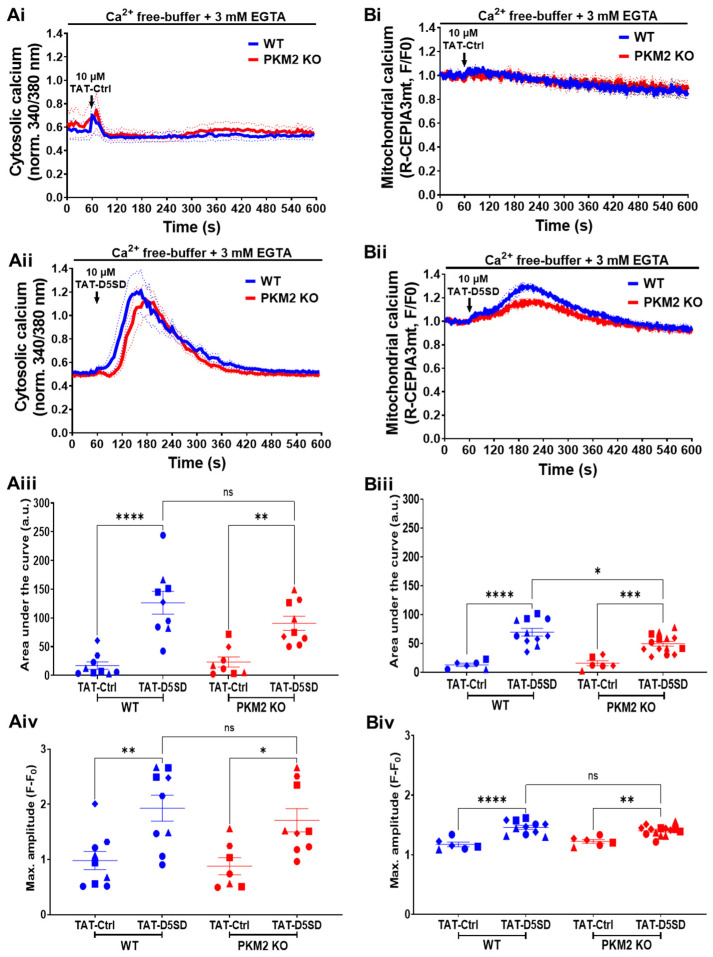
The TAT-D5SD peptide induces cytosolic and mitochondrial Ca^2+^ signals in HeLa WT and HeLa PKM2 KO cells. Time course of the cytosolic Ca^2+^ responses elicited by TAT-Ctrl (**Ai**) or TAT-D5SD (**Aii**) in HeLa WT (blue) and HeLa PKM2 KO (red) cells. The single-cell Ca^2+^ signals were measured in the absence of extracellular Ca^2+^ in cells loaded with Fura-2 AM. As indicated by the arrow, 10 μM of peptide was added after 60 s of baseline measurement. The curves are presented as the mean (bold line) ± SEM (dashed line) values of five independent experiments, each performed on 1–3 coverslips/experiment, with >35 cells/coverslip. (**Aiii**) The quantification of the area under the curve and (**Aiv**) maximal amplitude of the Ca^2+^ signals shown in Ai and Aii. Statistical difference was analyzed using a one-way ANOVA, followed by Tukey’s post hoc test (* *p* < 0.05; ** *p* < 0.01; **** *p* < 0.0001). The time course of mitochondrial Ca^2+^ uptake elicited by TAT-Ctrl (**Bi**) or TAT-D5SD (**Bii**) in HeLa WT (blue) and HeLa PKM2 KO (red) cells transfected with R-CEPIA 3mt. After 48 h, single cells’ mitochondrial Ca^2+^ signals were measured via fluorescence microscopy in the absence of extracellular Ca^2+^. As indicated by the arrow, 10 μM of the peptide was added after 60 s of baseline measurement. Mitochondrial Ca^2+^ uptake was normalized to baseline fluorescence. The curves are presented as the mean (bold line) ± SEM (dashed line) values of four independent experiments, each performed on 1–4 coverslips/experiment, with >10 cells/coverslip. (**Biii**) The quantification of the area under the curve and (**Biv**) the maximal amplitude of the mitochondrial Ca^2+^ uptake, shown in Bi and Bii as mean ± SEM values. Statistical difference was analyzed using a one-way ANOVA followed by Tukey’s post hoc test (* *p* < 0.05; ** *p* < 0.01; *** *p* < 0.001; **** *p* < 0.0001).

While the control peptide (TAT-Ctrl, 10 µM) did not evoke any Ca^2+^ signal in the cytosol (Figure 5Ai), the TAT-D5SD peptide (10 µM) induced a large, transient Ca^2+^ signal (Figure 5Aii). As extracellular Ca^2+^ was absent, the Ca^2+^ signal originated from the intracellular Ca^2+^ stores. Moreover, we previously demonstrated that the TAT-D5SD peptide did not evoke any cytosolic Ca^2+^ signal in HeLa 3KO cells which were completely devoid of IP_3_Rs [15]. However, the TAT-D5SD peptide evoked a similar increase in cytosolic Ca^2+^ in the HeLa PKM2 KO cells as in the HeLa WT cells, suggesting that TAT-D5SD acts in a PKM2-independent fashion (Figure 5Aii–Aiv).

Further experiments were performed to ascertain whether the TAT-D5SD peptide could generate Ca^2+^ signals in the mitochondria. The TAT-D5SD peptide (10 µM) caused mitochondrial Ca^2+^ uptake in both the HeLa WT and HeLa PKM2 KO cells, while the TAT-Ctrl peptide had no effect (Figure 5Bi,Bii). Nevertheless, the mitochondrial Ca^2+^ uptake caused by TAT-D5SD was significantly reduced in the HeLa PKM2 KO cells compared to the HeLa WT cells (Figure 5Biii). We speculate that the lower TAT-D5SD-evoked mitochondrial Ca^2+^ rise in the HeLa PKM2 KO cells was due to a reduction in ER-mitochondria contact sites.

The finding that the TAT-D5SD peptide acts very similarly in both HeLa WT and HeLa PKM2 KO cells is unexpected and led us to investigate the effects of the peptide on the induction of cell death in those same cells.

### 3.4. TAT-D5SD Increases the Number of Apoptotic HeLa WT and HeLa PKM2 KO Cells

**Figure 6:**
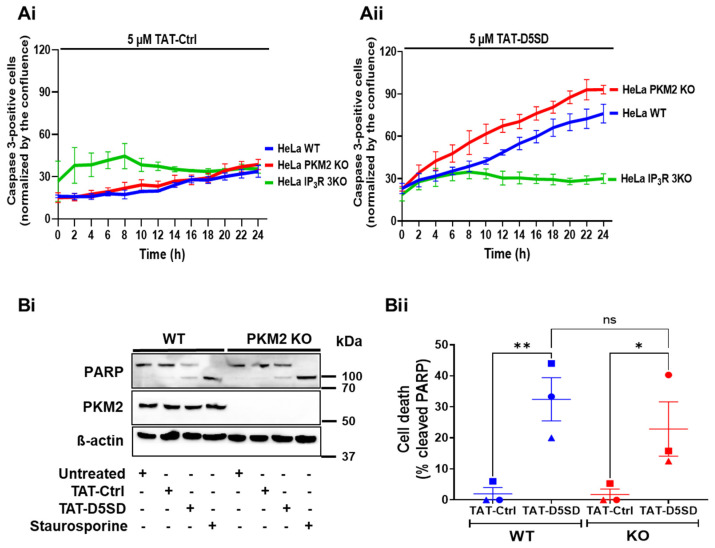
The TAT-D5SD peptide increases the number of apoptotic HeLa WT and HeLa PKM2 KO cells. The time course of the activation of caspase 3 by TAT-Ctrl (**Ai**) or TAT-D5SD (**Aii**) in HeLa WT (blue), HeLa PKM2 KO (red), and HeLa 3KO (green) cells. The cells were loaded with NucView^®^488 caspase-3 substrate (1 µM) in the presence of the TAT-Ctrl or TAT-D5SD peptide (5 μM). The cells were automatically photographed every 2 h using the IncuCyte ZOOM System, and the images were analyzed using the accompanying software. The results are displayed as the number of cells with activated caspase 3, normalized to the area confluence. Results are shown as the mean ± SEM values of three independent experiments, each performed on three wells/group. (**Bi**) PARP cleavage analysis performed in HeLa WT and HeLa PKM2 KO cells 12 h after treatment with TAT-D5SD (10 µM) or staurosporine (0.5 µM). After treatment, the cells were lysed, and the proteins were analyzed via Western blotting. A representative Western blot assessing uncleaved (top band) and cleaved (lower band) PARP, PKM2 and ß-actin is shown. β-actin was used as a loading control. Anti-PARP, anti-PKM1, and anti-ß-actin antibodies were diluted 1:1000. (**Bii**) The quantification of cleaved PARP in HeLa WT (blue) and HeLa PKM2 KO (red) cells. Data are represented as the mean ± SEM values of three independent experiments. Statistical difference was analyzed using a two-way ANOVA, followed by Tukey’s post hoc test (* *p* < 0.05; ** *p* < 0.01; ns = no statistical difference).

We previously demonstrated that treating cells with BIRD-2, a peptide disrupting Bcl-2:IP_3_R interaction, leads to apoptosis by eliminating the inhibitory effect of Bcl-2 on the IP_3_R, and thus leading to mitochondrial Ca^2+^ overload [31,32,33]. We therefore investigated whether TAT-D5SD, which elicits the release of Ca^2+^ into the cytosol as well as the mitochondria (Figure 5), will also result in cell death. Furthermore, to investigate the roles of PKM2 and the IP_3_R in this process, we compared the activation of caspase 3 in the HeLa WT and HeLa PKM2 KO cells and the HeLa 3KO cells that were completely devoid of IP_3_Rs [17] after treatment for up to 24 h with the TAT-Ctrl peptide (Figure 6Ai) or the TAT-D5SD peptide (Figure 6Aii). The TAT-D5SD peptide significantly increased the number of apoptotic HeLa WT and HeLa PKM2 KO cells, whereas the HeLa 3KO cells were highly resistant to the treatment. In contrast, the TAT-Ctrl peptide did not elicit a significant activation of caspase 3 in any of the cell lines.

We confirmed the effect of the TAT-D5SD peptide on the HeLa PKM2 KO cells by investigating PARP cleavage in the HeLa WT and HeLa PKM2 KO cells (Figure 6Bi,Bii). Both cell lines responded with PARP cleavage equally well to TAT-D5SD (10 µM) or staurosporine (0.5 µM) treatment for 12 h, while the TAT-Ctrl peptide was ineffective. Together, these results indicate the cytotoxic effect of TAT-D5SD is dependent on the activity of IP_3_R but does not rely on PKM2.

The observations that TAT-D5SD induced Ca^2+^ signals, as well as apoptotic cell death, in cells that either expressed PKM2 or were devoid of PKM2 means that one or more additional proteins are involved in the cellular responses to D5SD.

### 3.5. Examining the Potential Role of Lck and PKM1 in the Underlying Mechanism of Action of the TAT-D5SD Peptide

As the D5SD peptide was originally described to disrupt the Lck:IP_3_R1 interaction [34], it must be investigated whether the tyrosine kinase Lck is expressed in HeLa cells. Western blotting experiments, however, demonstrated that neither HeLa WT nor HeLa PKM2 KO cells expressed Lck (Supplementary Figure S2). Consequently, a potential interaction of the IP_3_R with Lck in those cells was not further scrutinized.

**Figure 7:**
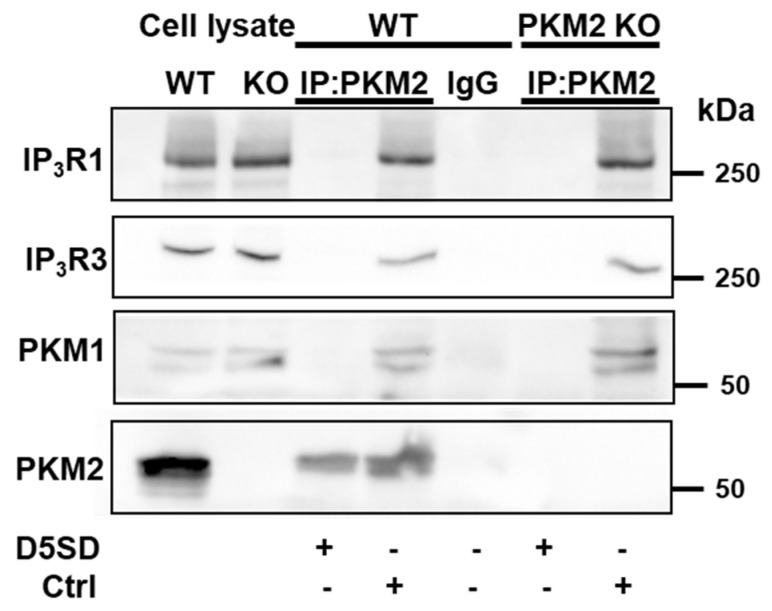
D5SD disrupts PKM2:IP_3_R and PKM1:IP_3_R interactions in HeLa cells. Representative co-immunoprecipitation experiment using an ant- PKM2 antibody (0.6 µg), performed in lysates from HeLa WT and PKM2 KO cells. This experiment was performed three times, each time using independently freshly prepared cell lysates incubated with Ctrl or D5SD peptides (200 μM). The samples were analyzed via Western blotting, using antibodies against IP_3_R1 (1:1000), IP_3_R3 (1:1000), PKM1 (1:1000), and PKM2 (1:1000). Total HeLa lysates were used as an input (10 µg). IP: immunoprecipitated; IgG: normal rabbit control IgG.

Further experiments demonstrated in HeLa WT cells that the anti-PKM2 antibody led, in addition to the immunoprecipitation of PKM2, IP_3_R1, and IP_3_R3, also to the precipitation of PKM1 (Figure 7). The epitope of the anti-PKM2 antibody is located in exon 10, coding for 56 a.a., which is specifically expressed in PKM2. However, exon 10 still shares 32 a.a. with exon 9, which is specifically expressed in PKM1, which allows for the recognition of the latter by the antibody. The immunoprecipitation of PKM1 was confirmed in the HeLa PKM2 KO cells, wherein IP_3_R1 and IP_3_R3 co-immunoprecipitated with it, suggesting that PKM1 can also interact with the IP_3_R.

**Figure 8:**
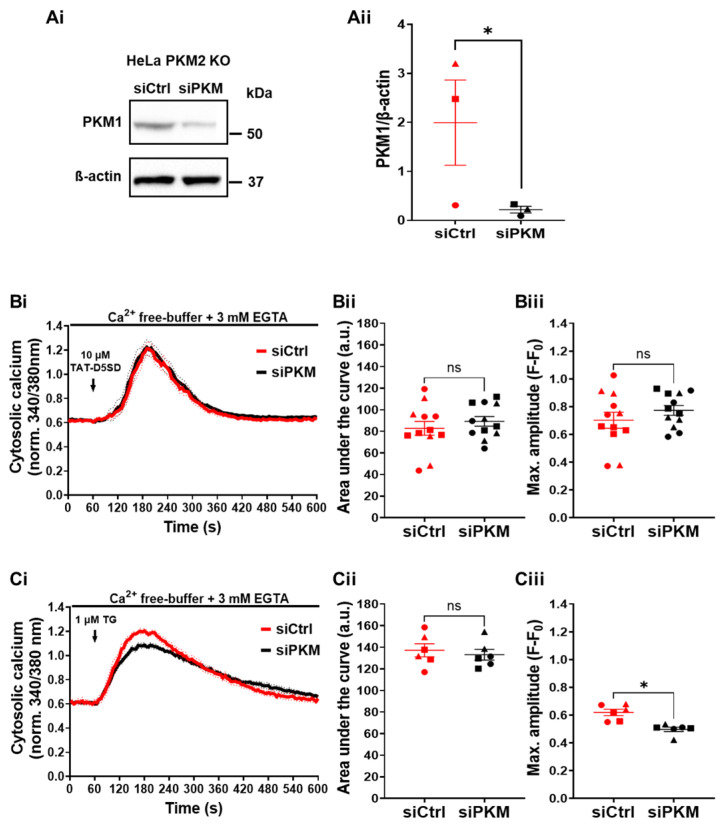
TAT-D5SD elicits cytosolic Ca^2+^ signals independently of PKM expression. (**Ai**) Representative Western blot of HeLa PKM2 KO cell lysate 48 h after transfection with either a siRNA targeting PKM (siPKM) or a non-target siRNA (siCtrl). β-actin was used as housekeeping protein. Anti-PKM1 and anti-ß-actin antibodies were diluted 1:1000. (**Aii**) The quantification of PKM1 from HeLa PKM2 KO cells transfected with siCtrl (red) and siPKM (black). Data are represented as the mean ± SEM values of three independent experiments. (**Bi**) The time course of the cytosolic Ca^2+^ responses elicited by TAT-D5SD in HeLa PKM2 KO cells transfected with siCtrl (red) or siPKM (black). The single-cell Ca^2+^ signals were measured in the absence of extracellular Ca^2+^ in cells loaded with Fura-2 AM after 48 h of transfection. As indicated by the arrow, TAT-D5SD (10 μM) was added after 60 s of baseline measurement. The curves are presented as the mean (bold line) ± SEM (dashed line) values of three independent experiments, each performed on four coverslips/experiment with >15 cells/coverslip. (**Bii**) The quantification of the area under the curve and (**Biii**) the maximal amplitude of the Ca^2+^ signals shown in Bi. Significance was analyzed using Student’s *t*-test (* *p* < 0.05; ns = no statistical difference). (**Ci**) The time course of the cytosolic Ca^2+^ responses uncovered by thapsigargin (TG) in HeLa PKM2 KO cells transfected with siCtrl (red) or siPKM (black). Single-cell Ca^2+^ signals were measured in the absence of extracellular Ca^2+^ in cells loaded with Fura-2 AM after 48 h of transfection. As indicated by the arrow, TG (1 μM) was added after 60 s of baseline measurement. The curves are presented as the mean (bold line) ± SEM (dashed line) values of Three independent experiments, each performed on two coverslips/experiment with >5 cells/coverslip. (**Cii**) The quantification of the area under the curve and (**Ciii**) the maximal amplitude of the Ca^2+^ signals shown in Ci. Significance was analyzed using Student’s *t*-test (* *p* < 0.05; ns = no statistical difference).

To investigate whether the effect of TAT-D5SD on Ca^2+^ handling in HeLa PKM2 KO cells was due to the dissociation of PKM1 from the IP_3_R, we performed an siRNA-mediated knockdown of PKM1 in the HeLa PKM2 KO cells. Using this approach, PKM1 levels were reduced by 86% (Figure 8Ai,Aii). In spite of this strong decrease in the PKM1 expression level, the TAT-D5SD peptide induced a similar Ca^2+^ release as in the HeLa PKM2 KO cells in which PKM1 was not downregulated (Figure 8Bi–Biii). As the Ca^2+^ store content measured after application of thapsigargin (1 µM) was unaltered in the PKM1 knockdown HeLa cells (Figure 8Ci–Ciii), these data strongly suggest that the action of the TAT-D5SD peptide on Ca^2+^ handling and apoptotic cell death are independent of its interaction with either PKM1 or PKM2.

## 4. Discussion

At present, more than 150 different proteins have been described to interact with one or more of the IP_3_R isoforms, leading to the formation of large multiprotein complexes [6,7]. The proteins participating in IP_3_R complexes can perform various functions, including stimulating or inhibiting IP_3_R activity, scaffolding the IP_3_R to the cytoskeleton, linking regulatory proteins to the IP_3_R, or transmitting downstream signals after being activated by the IP_3_R.

Interestingly, PKM2 recently joined the group of IP_3_R-interacting proteins [14,15]. PKM2 is hereby only the second metabolic enzyme shown to interact with the IP_3_R, the other being glyceraldehyde-3-phosphate dehydrogenase (GAPDH), which is also a glycolytic enzyme [35]. The stimulatory effect of the latter was explained by its localized production of NADH, which can activate the adenine-nucleotide-binding site(s) on the IP_3_R [36].

PKM2 is, in a certain way, reminiscent of GAPDH as it is also a tetrameric enzyme involved in glycolysis, is upregulated in cancer, and has numerous effects unrelated to its metabolic role, including in transcriptional regulation [10,12]. Moreover, in pancreatic ductal adenocarcinoma, the PKM2 in the glycolytic metabolon was shown to be associated with the plasma membrane and the resulting production of ATP is important to guarantee a sufficient supply of ATP to the plasma membrane Ca^2+^ ATPase in order to avoid cytotoxic Ca^2+^ overload, e.g., in conditions of severe hypoxia [37].

The previously obtained results concerning PKM2 and the IP_3_R highlight two different but complementary actions. On one hand, it was shown that PKM2 methylation decreases IP_3_R expression in breast cancer cells, though it also favored its interaction with the IP_3_Rs [14]. On the other hand, we demonstrated that PKM2 interacts with the various IP_3_R isoforms in several cell types, including B and T cells, and this leads to a suppression of its Ca^2+^-release activity [15]. To further investigate the role of the PKM2:IP_3_R interaction in physiological cell signaling, we developed a HeLa PKM2 KO cell line via the CRISPR/Cas 9 technique (Figure 1). A consequence of the loss of PKM2 was that the PKM1 isoform, which is not detectable in HeLa WT cells, was markedly upregulated. Such an upregulation of PKM1 was also observed in PKM2 KO fibroblasts [38], PKM2 KO keratinocytes [39], and the PKM2 KO breast cancer cell lines MCF7 and MDA-MB-231 [14]. In the latter cell type, however, the expression levels of the isoforms IP_3_R1 and IP_3_R3 were increased, while in the HeLa PKM2 KO cell line, we observed no changes in the IP_3_R nor in SERCA2 levels (Figure 1B).

In HeLa PKM2 KO cells, we observed an increased agonist-induced Ca^2+^ release in comparison to HeLa WT cells (Figure 2A). This is in full agreement with the previously described inhibitory effect of PKM2 on the IP_3_R [15]. Interestingly, at a high agonist concentration, eliciting similar cytosolic [Ca^2+^] increases in HeLa WT and PKM2 KO cells (Supplementary Figure S1), the loss of PKM2 evoked a decline in mitochondrial Ca^2+^ uptake (Figure 2B). The reasons for this decline must therefore be due to changes at the level of the mitochondria. We observed that the HeLa PKM2 KO cells displayed an increased number of mitochondria yet were smaller and less branched (Figure 3A). These mitochondrial changes converge with previous reports stating that PKM2 enhances mitochondrial fusion while inhibiting mitochondrial fission [40,41,42]. Moreover, in C2C12 myoblasts, it was previously reported that increased mitochondrial fusion correlated with higher mitochondrial Ca^2+^ uptake, while less mitochondrial Ca^2+^ uptake was observed upon mitochondrial fission [43]. Furthermore, the HeLa PKM2 KO cells contained fewer ER-mitochondria contact sites (Figure 3B), which may underlie the decreased ER-mitochondria Ca^2+^ transfer observed both in cells exposed to ATP or to TAT-D5SD. Paradoxically, in the HeLa PKM2 KO cells, the mitochondria were more polarized (Figure 4A,B), which theoretically would provide a larger driving force for the influx of Ca^2+^ to the mitochondria, but apparently this larger driving force cannot fully compensate for the decreased number of ER-mitochondria contact sites. ATP production in the HeLa PKM2 KO cells was only slightly affected (Figure 4C). On one hand, due to the upregulation of PKM1 in the PKM2 KO cells, ATP production due to glycolysis only decreased by 19%. On the other hand, as PKM2 drives metabolism away from OXPHOS via various mechanisms [42], in its absence, mitochondrial ATP production even slightly increased, although not at a sufficient level to fully compensate for the decrease in the production of ATP due to glycolysis.

As PKM2 KO affects the biology of cells at multiple levels, we opted to apply TAT-D5SD, a cell-permeable peptide previously reported to disrupt the PKM2:IP_3_R interaction [15]. This peptide corresponds to a.a. 2078–2098 of IP_3_R1 [34] and is located in its ARM3 domain [44], in close proximity to a crucial Ca^2+^-binding site for IP_3_R activation [45]. The cell-permeable form of the peptide, TAT-D5SD, at a concentration of 10 µM, induced a strong Ca^2+^ signal in the HeLa WT cells (Figure 5A). As the cells were surrounded by a Ca^2+^-free medium containing 3 mM of EGTA, the Ca^2+^ signal originated from intracellular Ca^2+^ stores and can be explained by a sensitization of the IP_3_R to basal IP_3_ concentrations, occurring when the D5SD peptide causes the dissociation of PKM2 from the IP_3_R. However, an identical response was observed in the HeLa PKM2 KO cells. A similar behavior was observed in the mitochondria, although the latter Ca^2+^ signal was smaller in the HeLa PKM2 KO cells than in the HeLa WT cells (Figure 5B), potentially due to the decreased ER-mitochondria contact in the HeLa PKM2 KO cells.

As excessive mitochondrial Ca^2+^ uptake is related to the induction of apoptotic cell death [26,30,46], we investigated the effect of treating the TAT-D5SD peptide on apoptosis. As markers for apoptosis, we used caspase 3 activity and PARP cleavage (Figure 6). The results indicated that the TAT-D5SD peptide induced apoptosis at a very similar level in the HeLa PKM2 WT and PKM2 KO cells. Notwithstanding the lower mitochondrial Ca^2+^ uptake observed in the HeLa PKM2 KO cells (Figure 5B), apoptosis progression was not slower than in the HeLa WT cells. This is not surprising as PKM2 suppresses apoptosis [47], e.g., via phosphorylation and the stabilization of anti-apoptotic Bcl-2 [48]. No apoptosis induction, however, occurred in the HeLa 3KO cells that lacked all three IP_3_R isoforms [17]. These results therefore indicate that the D5SD peptide acts in an IP_3_R-dependent way, though PKM2 expression is dispensable. Although the D5DS peptide was originally described as a peptide corresponding to the interaction site on IP_3_R1 for the tyrosine kinase Lck [34], this protein was neither detected in the HeLa WT nor in HeLa PKM2 KO cells (Supplementary Figure S2), making a role for it highly unlikely.

Taken together, these data strongly suggest the involvement of still another protein that could regulate the IP_3_R and interact with the D5SD peptide. As the HeLa PKM2 KO cells displayed upregulated PKM1 levels, we investigated the possibility that PKM1 also interacts with the IP_3_R (Figure 7). Although in breast cancer cells, PKM1 did not appear to interact with the IP_3_R [14], in our experimental analyses, both IP_3_R1 and IP_3_R3 co-immunoprecipitated with PKM1. To investigate the role of PKM1 underlying TAT-D5SD effects on cells, we effectively downregulated PKM1 in HeLa PKM2 KO cells using siRNA (Figure 8). Despite this downregulation, the TAT-D5SD peptide remained as active in releasing Ca^2+^ from intracellular stores as the cells treated with a control siRNA (Figure 8). This effect cannot be due to a higher level of Ca^2+^ store loading, as, when measured, the Ca^2+^ stores treated with siRNA against PKM1 contained the same Ca^2+^ content as to the cells treated with control siRNA. As such, TAT-D5SD evokes cell death through a mechanism involving IP_3_Rs though targeting a protein different than PKM2, PKM1, and Lck. As PKM2 interacts with and stabilizes VDAC3 [49] and the IP_3_R is coupled via GRP75 to VDAC1 in the outer mitochondrial membrane [50], these proteins come to mind as possible candidates. This is fully compatible with the idea that the IP_3_R forms a multi-protein complex interacting with mitochondrial VDAC [30,51]. Alternatively, we cannot fully exclude that TAT-D5SD peptide directly impacts IP_3_R gating through homotypic interactions or by interfering with interdomain interactions, eventually resulting in IP_3_R channel opening.

## 5. Conclusions

Taken together, the present study extends the findings concerning the regulation of the IP_3_R by the metabolic enzyme PKM2 and indicates a differential effect of PKM2 on cytosolic and mitochondrial Ca^2+^ handling. The latter is due to the effects of PKM2 not only on mitochondrial metabolism but also on mitochondrial structure and dynamics. Finally, using the TAT-D5SD peptide that corresponds to amino acids 2078-2098 of IP_3_R1 and which disrupts the PKM2:IP_3_R complex, we observed that the effects of this peptide on Ca^2+^ handling and apoptosis are dependent on the IP_3_R but do not necessitate the presence of PKM2. As the presence of PKM1 and the tyrosine kinase Lck are also not needed, our results strongly suggest that one or more other proteins interact with the IP_3_R at the same site as PKM2 and Lck and that amino acids 2078–2098 are located at or near to a crucial regulatory site on the IP_3_R.

## Data Availability

All relevant data are presented in the manuscript and the Supplementary Materials. The full-length immunoblots are included as Supporting Information files. The underlying data are available via the KU Leuven RDR (research data repository) at https://rdr.kuleuven.be (accessed on 23 October 2023) with https://doi.org/10.48804/7GEY30.

## Supplementary Materials

The following supporting information can be downloaded at: https://www.mdpi.com/article/10.3390/cells12212527/s1, Figure S1: Time-course of the Ca^2+^ responses evoked by 10 μM of ATP in HeLa WT and HeLa PKM2 KO cells; Figure S2: Western blot showing that HeLa WT and PKM2 KO cells do not express Lck.
