# Supplementary material for: The Complex Effects of PKM2 and PKM2:IP3R Disruption on Intracellular Ca2+ Handling and Cellular Functions
Source: Cells. 2023 Oct 26;12(21):2527. doi: 10.3390/cells12212527 (PMC10647343; doi:10.3390/cells12212527)
Supplement: Supplementary file 1 [file cells-12-02527-s001.zip › cells-2649211-supplementary.pdf]

Supplementary Materials

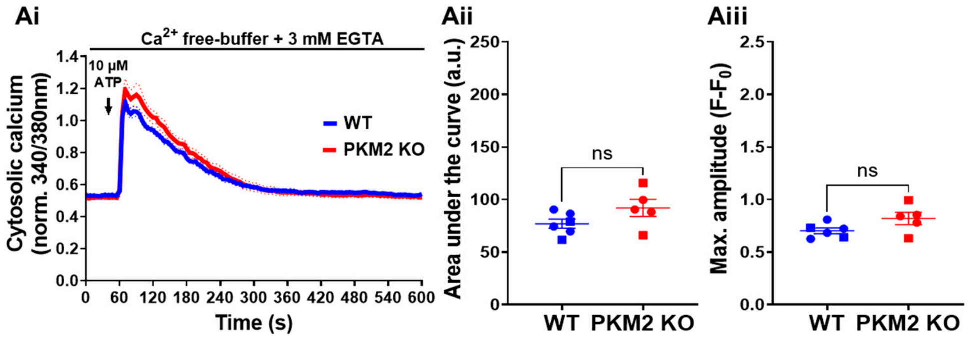

Figure S1. (Ai) Time-course of the Ca^{2+} responses evoked by 10 μM ATP in HeLa WT (blue) and HeLa PKM2 KO (red) cells. The single-cell Ca^{2+} signals were measured in the absence of extracellular Ca^{2+} in cells loaded with Fura-2 AM. As indicated by the arrow, 10 μM ATP was added after 60 s of baseline measurement. The curves are presented as the mean (bold line) ± SEM (dashed line) of 2 independent experiments, each performed on 2-4 coverslips/experiment, >45 cells/coverslip. (Aii) Quantification of the area under the curve and (Aiii) maximal amplitude of the Ca^{2+} signals induced by 10 μM ATP, shown in Ai. Significance was analyzed with Student's t-test (ns = no statistical difference).

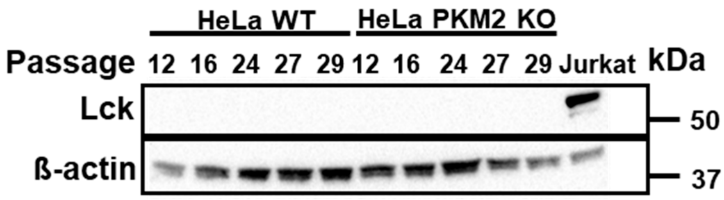

Figure S2. Western blot showing that HeLa WT and PKM2 KO cells do not express Lck. Cell lysates from 5 different passages are shown. β-actin was used as loading control. Anti-Lck antibody was diluted 1:1000. A Jurkat T cell lysate was used as positive control.
